# The Platform Technology Approach to mRNA Product Development and Regulation

**DOI:** 10.3390/vaccines12050528

**Published:** 2024-05-11

**Authors:** John H. Skerritt, Carolyn Tucek-Szabo, Brett Sutton, Terry Nolan

**Affiliations:** 1 Faculty of Medicine, Dentistry and Health Sciences, University of Melbourne, Melbourne, VIC 3010, Australia; t.nolan@unimelb.edu.au; 2 Moderna Australia, 101 Collins St, Melbourne, VIC 3000, Australia; carolyn.tucekszabo@modernatx.com; 3 CSIRO Health and Biosecurity, Research Way, Clayton, VIC 3168, Australia; brett.sutton@csiro.au; 4 Peter Doherty Institute for Infection and Immunity, 792 Elizabeth St, Melbourne, VIC 3000, Australia

**Keywords:** mRNA, vaccine development, drug development, regulation

## Abstract

mRNA-lipid nanoparticle (LNP) medicinal products can be considered a platform technology because the development process is similar for different diseases and conditions, with similar noncoding mRNA sequences and lipid nanoparticles and essentially unchanged manufacturing and analytical methods often utilised for different products. It is critical not to lose the momentum built using the platform approach during the development, regulatory approval and rollout of vaccines for SARS-CoV-2 and its variants. This review proposes a set of modifications to existing regulatory requirements for mRNA products, based on a platform perspective for quality, manufacturing, preclinical, and clinical data. For the first time, we address development and potential regulatory requirements when the mRNA sequences and LNP composition vary in different products as well. In addition, we propose considerations for self-amplifying mRNA, individualised oncology mRNA products, and mRNA therapeutics. Providing a predictable development pathway for academic and commercial groups so that they can know in detail what product characterisation and data are required to develop a dossier for regulatory submission has many potential benefits. These include: reduced development and regulatory costs; faster consumer/patient access and more agile development of products in the face of pandemics; and for rare diseases where alternatives may not exist or to increase survival and the quality of life in cancer patients. Therefore, achieving consensus around platform approaches is both urgent and important. This approach with mRNA can be a template for similar platform frameworks for other therapeutics and vaccines to enable more efficient development and regulatory review.

## 1. Introduction

mRNA vaccines have been instrumental in significantly reducing global mortality and morbidity from SARS-CoV-2 infection [1]. With the successful administration of mRNA vaccines to billions of people, much has been learned about safely accelerating regulatory review and approval processes for subsequent iterations of the initial vaccine and regulatory flexibility using a platform technology approach. The need to optimise regulatory pathways for new or emergent pandemic threats (Disease X) has focussed international attention on better and faster processes. A wide range of other mRNA vaccines and therapeutics are in advanced stages of development, including for infectious diseases, oncology and rare diseases. There are also potential safety advantages for mRNA therapeutics over gene editing therapies because there is no genome integration or modification of the genome. 

mRNA products can be considered platform technology because their development and manufacturing process is consistent, with similar noncoding mRNA sequences and lipid nanoparticle technologies often utilised [2]. This review summarises how existing experience with manufacturing, quality control, preclinical, and clinical development of mRNA products could be utilised by product developers. It describes the hierarchy of products to which a platform technology approach can be applied, and how the authors propose that bridging and comparability studies could be utilised for these products. 

Special considerations for self-amplifying mRNAs or when the LNP component is modified, and applications of mRNA platform technology in the development and regulatory review of mRNA therapeutics and individual neoantigen therapies, are also discussed. The review is based on principles developed by a diverse group of experts which can inform subsequent regulatory guidance. It provides the first compilation of potential regulatory requirements for mRNA products from a chemistry manufacturing and controls (CMC), as well as a preclinical and clinical platform, perspective.

## 2. Streamlined Regulatory Pathways for mRNA Platform Technologies Are Possible

Regulators already have the legal frameworks to review novel mRNA products. However, there are mutual benefits for developers, industry, regulators, and patients awaiting access to innovative therapies if platform approaches for these products are agreed upon and implemented. Sufficient experience has been gained to build confidence in mRNA technology. There is a significant opportunity to build upon the experience with COVID-19 vaccines, including the several modified vaccines targeted at later variants. 

This issue is important because many of the mRNA products already in clinical development address areas of significant unmet medical need—including viral diseases of global significance, metabolic diseases, and a range of serious cancers. It is urgent because regulatory submissions for non-COVID mRNA products have already been made to regulators worldwide during 2023, with several more submissions to be made in 2024/2025. mRNA vaccines offer the most effective means of rapidly developing a vaccine against the next pandemic pathogen—an objective prioritised in CEPI’s (Coalition for Epidemic Preparedness Innovations) preparedness for ‘Disease X’ [3]. 

A ‘drug platform’ refers to the similarities across multiple products with respect to molecular structure and product composition, non-clinical attributes, manufacturing process and product quality attributes. The ability to consider a group of mRNA products as a platform depends upon the extent of information available through quality, safety, and efficacy assessments of preceding products. This information is critical to understanding the impacts that any significant difference in a new mRNA product would have. In turn, this would support regulators in determining the type of information they should request from sponsors. 

The process of development and manufacturing of mRNA products has been reviewed extensively [4,5,6,7]. It is similar for very different diseases and conditions, and therefore is justifiably classified as a platform technology [8,9]. This process of identifying optimised protein followed by mRNA design and synthesis is essentially repeated to create other medicines and vaccines. The mRNA is produced in a standardised reaction, with different manufacturers using a similar protocol regardless of the coding sequence of the mRNA (typically the same process with recipe changes). Other parts of the mRNA used in the vaccine or medicine from a given manufacturer are often largely the same in different products. The same or similar lipid nanoparticle technology (used to deliver the vaccine or medicine into the target cells) can be used for different products. 

While there are broader classes of RNA vaccines and therapeutics under development, and a range of mRNA delivery technologies currently being researched, this review only considers mRNA-lipid nanoparticle (LNP) and self-amplifying mRNA (sa-mRNA) drug products. This is because these represent the largest pipeline of mRNA vaccines and therapeutics under development. 

## 3. Regulators’ Experience and Expectations with Platform Technologies 

Section 2503 of the 2022 US Food and Drug Omnibus Reform Act required the US Food and Drug Administration (FDA) to create a designation program for “platform technologies”. Platform technologies are defined as technologies that have the potential to be incorporated in, or used by, more than one drug or biological product and are reasonably likely to make the drug development or manufacturing process and the review process more efficient. If FDA designates a particular platform technology, FDA “*may expedite the development and review of any subsequent application submitted under Section 505(b) of [the Food, Drug, and Cosmetic] Act or Section 351(a) of the Public Health Services Act for a drug that uses or incorporates the platform technology”.* Sponsors may also “reference or rely upon data and information” from a previous application from the same sponsor for a drug or biological product that incorporates or uses the same platform technology. 

The European Medicines Agency (EMA) in 2023 conducted a consultation on development of a guideline on Module 3 (quality) aspects of mRNA vaccines [10]. The EMA statement on updating vaccines in response to new SARS-CoV-2 variants [11] also states “*using a platform approach, as already experienced for adapted mRNA vaccines in 2022, is considered acceptable to approve strain change variations. Approvals can be based on manufacturing/quality and non-clinical data only, provided the vaccine platform can demonstrate predictability of clinical immunogenicity and reactogenicity. Such clinical data can be based on different variants of concerns that have been previously investigated”.* With the recent adoption of the European Parliament of new General Pharmaceutical Legislation, it is also anticipated that the new Directive will provide a legal definition of platform technology including platform technology master files.

The WHO Expert Committee on Biological Standardization has defined platform technology for prophylactic mRNA vaccines [12], while several International Conference on Harmonization of technical requirements for registration of pharmaceuticals for human use guidelines also address platform technologies. The WHO also recently defined a platform technology in the context of prophylactic mRNA vaccines for infectious diseases as “*a group of technologies used as a base upon which other applications, processes or technologies are developed*”. WHO stated that the term can be applied to a particular drug-delivery system, such LNPs containing the mRNA, where identified lipids, concentrations, and methods of preparation and purification, among other things, are used. 

### Utilisation of Platform Technology in mRNA Regulatory Submissions and Review

Regulators have historically used information across products for expediting the review of new products. This type of extrapolation, usually referred to as leveraging prior knowledge [13], is also frequently employed during drug development as a product’s composition or manufacturing evolves. One approach is through use of comparability protocols, where a change that may affect key quality attributes of a drug product related to its safety or efficacy may be implemented without requiring full *de novo* evaluation. 

In general, platform components that can be leveraged in regulatory submissions for subsequent products include elements that are integral to the composition of the platform product and have been utilised in successive products without any impacts on safety, manufacturing quality, or efficacy [14,15,16,17,18]. These can include mRNA attributes, such as codon usage and optimisation, regulatory sequences in untranslated regions, and LNP composition, if these are largely unchanged from those in the original product. In addition, significant parts of the non-clinical part of the submission should be able to be leveraged such as non-clinical safety data. However, data from specific clinical trials are typically required to be submitted for each new product, and data from related mRNA-LNP platform products can support and be leveraged in investigations around posology, pharmacokinetic, and pharmacodynamic characteristics and potentially the prediction of reactogenicity and other adverse events. 

The extent to which platform approaches can be utilised in regulatory submissions depends on the degree of similarity between the structural composition, intended effect, manufacturing process and product quality, and proposed context of use between mRNA products. To effectively utilise platform approaches in regulatory submissions, sponsors are required to have sufficient information to understand the relevance of differences between a new mRNA product and preceding platform products. It will also be necessary to justify to the regulatory agency as to why particular comparability and bridging exercises are appropriate. 

## 4. mRNA-LNP Products in the Market or under Late-Stage Clinical Development

Many of the mRNA products in clinical development address significant unmet medical need, including infectious disease, oncology, and rare disease applications. mRNA vaccines may also offer the most effective means of rapidly developing a vaccine against the next pandemic pathogen. mRNA technology can potentially improve on vaccines that are suboptimal. The technology also enables a nimble response to both unmet medical needs and rapidly emerging threats. If a genetic basis for a rare disease can be identified that involves under- or incorrect expression of a particular enzyme or other protein, mRNA technology can also be employed. 

As of January 2024, the only human mRNA products with regulatory approval were a range of vaccines against SARS-CoV-2 (and variants). For brevity, these are not summarised here. In mid-2023 an RSV (respiratory syncytial virus) mRNA product was submitted for review to major regulators. It is anticipated that during 2024 and 2025 several companies will make regulatory submissions for a number of mRNA products. Products in phase 1–3 clinical trials from the major companies as of 1 May 2024 are listed in Table 1. There are also over 250 entries under “mRNA” on clinicaltrials.gov for phase 2 or 3 clinical trials. Examples of conditions targeted in phase 2 trials listed in the database include:

- Respiratory viruses—SARS-CoV-2, respiratory syncytial virus, seasonal influenza.
- Other infectious diseases—HIV, Lyme disease, cytomegalovirus.
- Cancers—malignant melanoma, uveal melanoma, lymphoma, solid tumours, pulmonary osteosarcoma, prostate cancer, head and neck cancers, gastric cancer, pancreatic cancer, ovarian cancer, biliary tract cancer.
- Rare or metabolic diseases—methylmalonic acidemia, ornithine transcarbamylase deficiency, phenylketonuria, propionic aciduria, primary ciliary dyskinesia.

An analysis of data in clinicaltrials.gov as of 1 April 2023 [19] found there were 416 trials involving mRNA products, of which 20% involved diseases other than SARS-CoV-2. The diseases were as above, but trials for breast cancer, glioblastoma, hepatitis B, and tuberculosis were also identified. Some oncology trials, e.g., of individualised neoantigen therapies take advantage of the ability to rapidly synthesise mRNA sequences specific for individual patients. A number of other mRNA products also target diseases for which there are either no effective therapies or inadequate vaccines, including HIV, TB, malaria, cytomegalovirus, Zika, Nipah, and Lyme, as well as rare metabolic diseases [20,21,22,23,24,25,26,27,28].

## 5. Data Requirements for mRNA Product Development and Regulatory Submissions

Regulatory submissions follow a standardised structure internationally, known as the Common Technical Document (CTD). The dossier is divided into five main modules:

- Module 1—Administrative information and prescribing information.
- Module 2—Overviews and summaries of Modules 3–5.
- Module 3—Quality (chemistry, manufacturing, and controls) reports for the drug substance (mRNA) and (finished) drug product.
- Module 4—Non-clinical reports (pharmacology, pharmacokinetics, and toxicology).
- Module 5—Clinical study reports (including biopharmaceutic studies, human pharmacokinetic and pharmacodynamic studies, clinical trial (efficacy and safety) studies, and post-marketing experience).

### 5.1. Quality (Chemistry, Manufacturing, and Controls (CMC)) 

While the basic principles of product quality and their regulatory requirements are similar for mRNA and other therapeutic products, there are several unique CMC attributes to mRNA products. Many mRNA platform efficiencies emerge directly from the in-depth understanding of data and information gathered that links product quality and manufacturing processes [29]. The WHO Expert Committee on Biological Standardization [12] have provided some considerations on CMC requirements for mRNA vaccines. To establish an efficient process for developing a regulatory dossier for a new mRNA product within a platform or to support evaluation of changes to a product as part of the mRNA platform, a sponsor could create a proprietary master comparability protocol (master file) for that product. This would include specific tests, analytical procedures, and acceptance criteria for specified changes expected over the life cycle of the mRNA platform product.

Details on the method of manufacture of the mRNA, LNPs, and final drug product is required, including data on the critical quality attributes of the intermediates and final products, in process controls, and in the sterilisation procedures used. The starting materials that are subject to quality control points include linearised plasmid DNA templates from plasmid or in vitro DNA transcription methods, nucleotides, and cap analogues or methyl-donating molecules, as well as buffers, column resins, and enzymes [17,30]. The cell bank system for bacterial culture and amplification of plasmids requires testing for identity, stability, microbial purity, and freedom from contamination [31]. DNA plasmids require testing to confirm identity, purity, and integrity using DNA template sequencing. The methods of plasmid purification and their effectiveness need to be documented and validated. Specifications for critical quality attributes for the identity, purity, quantity, and physical state and safety of the bulk purified RNA must be established and justified. The tests at each stage for production of the drug substance need to be documented. The level of consistency of the mRNA capping and polyadenylation processes should be assessed and documented. Analysis must confirm sequence identity and integrity. Fragments and off-target (truncated, readthrough, or antisense) RNAs are considered impurities as they can stimulate an unwanted innate immune response. 

The process of mRNA encapsulation into LNPs needs to be documented and validated, including with information on the concentrations of different lipids, mRNA/lipid ratios, pH of buffers, encapsulation efficiency and flow rates during the encapsulation process. Release specifications for both the drug substance and drug product need to be documented. Product purity control strategies involve assessment of process-related and product-related impurities as well as other potential contaminants and methods to control them. 

The quality analytical testing of mRNA products may also be more complex, as it has attributes of both chemical medicines and biologicals. There is thus a significant focus on the control strategies for both the mRNA manufacturing process, as well as the need for detailed characterisation and release testing of the drug substance and, finally, filled mRNA-LNP product. However, with the platform nature of products, the submission can identify where the manufacturing processes used have been very similar or identical to predicate mRNA products. Several reviews of mRNA quality requirements and product testing have been published [4,12,16,29,30,31,32], while the United States Pharmacopeia [33] provides detailed experimental protocols for each testing method. 

While initial commercial production lots should be analysed using a comprehensive series of tests, with the platform nature of mRNA technology, for subsequent batches, a more limited series of tests akin to those required for vaccine lot or batch release may be able to be agreed upon with the regulatory agency, as well as for other products manufactured using a closely related platform. Tests are generally performed by reference to an in-house reference material which is a suitable, well characterised batch, and known from clinical trials to have the desired clinical effects. Tests that are typically required include mRNA sequence identity confirmation, RNA concentration, and intactness, purity, and safety. 

Potency testing of mRNA products [34,35] at release and during stability testing is expected by regulators. It is critical to demonstrate that the manufactured mRNA can express a complete encoded protein of the correct identity and that significant amounts of truncated or alternative sequences are not produced. Expression of these could produce new antigens that, if administered, could provide unwanted immune responses and adverse events [36]. A range of cell-based assays have been developed to assess levels of expression of target proteins. Evidence is required that they correlate with testing results from in vivo models in animals in pre-clinical studies, as potency depends both on effective mRNA translation in vivo and delivery to the appropriate tissues. Specific assays require development for each new type of expressed protein [34,37], and should measure a surrogate of the desired immunological response to the vaccine [35]. 

While potency assays based on expression of the target protein are necessarily candidate-specific, for related mRNA products produced within a given platform, it is expected that the assay used for the original product will be able to be adapted. Measurement of the mRNA-LNP target protein expression in eukaryotic cells may be a suitable surrogate measurement if it can be linked to potency (e.g., in vivo mouse or other model of immunogenicity). Test methods include expressed protein measurement using immunoblotting, cell staining, mass spectrometry, or flow cytometry using a specific monoclonal antibody to the target protein [20,21], although there are challenges with such assays when lower doses of vaccines are delivered using sa-mRNA technologies. The cellular site of protein accumulation, such as on the cell surface or entrapment in the endoplasmic reticulum, together with the tissue locations of protein expression, can have a large impact on potency and are often complex issues to measure empirically. 

Product characterisation testing will help assess which quality attribute(s) of platform products best correlate with potency (hence efficacy), as well as toxicity/reactogenicity, and can be considered clinically relevant attributes. It is possible to link attributes such as mRNA content, purity, and target protein expression to potential in vivo biological activity for a given disease. Different vaccine correlates of protection may require different modes of protein expression, such as cell surface expressed protein antigen for antibody-mediated protection. Once this has been demonstrated for a mRNA platform, future mRNA-LNP products of the same product family may also apply the same strategy to demonstrate potency. For some therapeutic targets, it may not be possible to link a product attribute to an in vivo activity, and a quantitative biological assay that measures the specific ability of the product to effect a given result will be required. 

A significant number of in-process controls and test results are required for LNPs. However, once tests are developed and results documented, if the same or highly similar LNPs are used for subsequent products, then the benefits of the platform approach apply. At the final stage of LNP synthesis, characterisation tests may include physical parameters (particle size, charge, lipid content, and lipid identity), compendial testing (bioburden, endotoxin, osmolality, and pH), and other quality testing (residual solvent impurities, lipid impurities). 

mRNA-LNP product characterisation tests include confirmation of the LNP size distribution, LNP surface characterisation, LNP charge and relative protein expression. In-process control tests may include the total RNA content, bioburden, endotoxin, and final release tests may include percent of mRNA encapsulation, mRNA purity, and amounts of remaining process-related impurities. For stability studies, comparability studies and re-use of test approaches can potentially be employed, although some regulators may want product-specific stability data to justify storage conditions and shelf-life [38]. A number of consecutive batches should be tested using the range of analytical methods listed above to check for consistency of manufacture. 

Good Manufacturing Practice (GMP) requirements apply to mRNA vaccines and therapeutics through the whole manufacturing process from starting materials to finished product, with quality control through the process being based in implementation of sound quality systems to enable manufacture of consistent lots of product [39]. However, if a new product is manufactured at a site that has been previously GMP-licensed or cleared/certified for manufacture of products containing very similar mRNAs and LNPs, a waiver could be requested by the sponsor to exempt the manufacturer of re-inspection provided adequate justification was provided. For example, a desk top audit that reviews the comparability of the process of manufacture of the new product could be conducted.

### 5.2. Non-Clinical Study Reports

While the platform technology approach can also be used here, a more extensive non-clinical and clinical assessment of products is typically required. The pharmacology Section will include information on the rationale for the selection of the target protein and how these relate to the mechanism of action of the vaccine or therapeutic. In cases when particular epitopes are selected for vaccine development, the rationale for the epitope selection approach should be documented [40]. The regulatory authority will typically require submission of the complete sequence of the open reading frame of the target antigen and the use of modifications to the mRNA and addition of other structural elements justified. Data on the extent and durability of the immune response including antibody titres, neutralising antibody, and cell-mediated immunity responses in pre-clinical studies should be obtained.

It is anticipated that pharmacokinetic studies will be required for mRNA therapeutics, but vaccines typically do not require regulatory pharmacokinetic or biodistribution studies [41]. This is because, for most vaccines which are administered intramuscularly, most of the dose remains in the muscle and the rest is eliminated through the lymphatic system. To date, regulators have required some pharmacokinetic and biodistribution studies for mRNA-LNP products [19], especially ancestral COVID-19 vaccines, although human pharmacokinetic studies were not required. Specific analytical studies were required to be developed and validated, and preclinical methods that can be used for biodistribution studies have been reviewed [42]. Greater experience across different mRNAs and LNPs make the platform approach fully applicable to biodistribution studies, however.

Separate animal pharmacokinetic studies were not required for the COVID-19 variant vaccines, as they utilised similar-sized mRNA and identical LNP, which is an example of regulatory acceptance of the platform approach (see Section 7.1). However, accumulation of as much evidence as possible on biodistribution and persistence of the mRNA-LNP, constituents and expressed protein will be important to support the establishment of a platform approach for products with similar mRNA-LNP characteristics and similar routes and frequencies of administration. Measurements should determine the extent to which the mRNA, LNP, and lipid components migrate away from the tissue into which the vaccine was administered, organs in which they distribute, and length of persistence. 

After establishing the biodistribution profile of an LNP, those nonclinical study data will be similar or identical for other products with the same LNP and can be re-used across programs. Data on other aspects of the mRNA-LNP’s pharmacokinetics (e.g., absorption, metabolism, and excretion) can also be re-used in subsequent regulatory submissions where the LNP is unchanged. Potential immune responses and reactogenicities can be predicted, to some extent, in nonclinical studies using reporter cell assays, while only some serious adverse events can be predicted. It is also important to assess immune activation and toxicities from both individual components and the combined vaccine or therapeutic. 

### 5.3. Application of the Platform Approach to Clinical Data 

With the exception of vaccines based on variants to a particular infectious disease for which a mRNA vaccine has been previously approved from the same manufacturer, and for which the non-coding regions of the mRNA have had minimal change, it is anticipated that regulators will require clinical trial data in other submissions for mRNA products. As for other therapeutic or vaccine products, regulatory submissions must describe the intended clinical use (target pathogen/disease and target population), and the rationale for selection of the target antigen (and thus the coding sequence used, e.g., for vaccines see [43]). For vaccines, apart from trial data on clinical vaccine efficacy against disease, data submitted for review should include data on the capability of the mRNA to trigger both antigen-specific responses and innate immune responses, as well as reactogenicity and any adverse events of special interest [44]. Re-use of earlier clinical data would be limited to changes within the same narrow classes of products (e.g., within COVID-19 or seasonal influenza or potentially mRNA-LNP products intended to treat genetic subsets of the same condition). 

More limited clinical evaluations of efficacy may be appropriate in some cases where there is understanding of the relationship, e.g., between immunogenicity and efficacy outcomes. Methods for assessment of humoral and cellular immunity to mRNA vaccines have been summarised [45]. For example, sponsors could rely upon surrogate measures of efficacy to demonstrate that updates to a prophylactic mRNA vaccine for seasonal strains or new variants of concern will have efficacy comparable to the previously approved products because the updated product will rely upon the same well-established mechanism of action. Seasonal influenza vaccine updates may be approved based upon relatively small immunogenicity studies or without human immunogenicity studies at all [46]. Subsequent mRNA platform products may similarly benefit from streamlined clinical data requirements. Identifying optimal dosages is difficult, but the maximum tolerated dosage for a given patient population, as determined for dosages of candidate vaccines, can form the basis of a platform approach for related vaccines. 

The platform nature of mRNA products may also allow for leveraging safety data across products to expand Phase 3 dosing for a new product earlier in development than may otherwise be allowed. In the context of an approved (or clinically well-studied) mRNA-LNP product, a combination of information about the disease, the biological effects of the product, and pharmacokinetic and pharmacodynamic data (or other kinds of clinical bridging data) in both populations may be sufficient to even leverage existing safety data for a new population (e.g., paediatric or pregnant or lactating patients).

## 6. Use of Comparability and Bridging Studies 

It may be necessary for the sponsor to perform clinical or nonclinical bridging and/or analytical comparability studies to establish whether any differences have potential impact on safety, quality, or efficacy of the product. It is advisable that the design of these studies and the data to be obtained is agreed between the sponsor and the regulatory agency before studies are conducted. Comparability protocols enable limited changes to a product to be considered in a regulatory context without requiring full de novo evaluation of the product’s safety and efficacy [14,15,16,17,18]. Long-standing examples include comparability studies for small molecule generic therapeutics, biosimilar comparability with the original monoclonal antibody therapeutic, and, more recently, for cell and gene therapeutics. 

The concepts of product similarity, predictive capacity of product attributes, assessment of the extent of difference, and inclusion of comparability assessment information can be used in regulatory submissions to describe the types of differences that would be expected between platform products. The degree of comparability can inform the extent to which information on existing processes, nonclinical and clinical data, and regulatory conclusions may be referenced for a new platform product. A comparability protocol [27] describes the specific tests and studies to be performed and the acceptance criteria to be achieved to demonstrate the lack of adverse effect of one or more proposed changes on product quality. The protocol should also include the analytical procedures to be used or reference thereto. However, specific mRNA comparability guidance requires development. Comparability guidance for advanced therapy medicinal products will be relevant [18]. Side-by-side testing of products in the same analytical run is the preferred approach for demonstrating comparability. The comparison of post-change data to historical data is not recommended, but is only acceptable if a side-by-side study is not possible. 

Bridging studies are required when a comparability assessment identifies important differences between platform products or where a new platform product has novel structural composition or manufacturing processes. In the context of manufacturing changes to an approved product, the USFDA has defined bridging studies as those “*performed to provide nonclinical or clinical data that allow…extrapolation of the existing data from the drug product produced by the current process to the drug product from the changed process”.*

Process comparability can be demonstrated through an assessment of manufacturing process controls against expected ranges or product acceptability ranges from the existing advanced mRNA product. For this comparability assessment, it is proposed that one post-change Process Performance Qualification (PPQ) lot (for the updated mRNA product) could be compared to the three pre-change PPQ lots (of the existing mRNA product) because the updated mRNA product is considered an additional replicate of the same platform process rather than a single lot from a new process. A sponsor may also potentially apply the same claimed shelf life and storage duration registered for the established mRNA platform. Confirmation could subsequently be obtained post-market, although until a larger number of products have been manufactured from a particular platform, regulators may require real-time stability from each product. 

Immunobridging is a well-established process for vaccines [41,47,48]. Once vaccine efficacy has been shown in a clinical trial conducted under one set of conditions, immunobridging has been widely used to infer vaccine efficacy for a different age or demographic group, different doses or dosing regimen or formulation, and variants or concomitant administration with other vaccines. When justified by data and on scientific principles, the use of immunobridging (based on an appropriate immune marker) can avoid the need to conduct another clinical endpoint efficacy trial. The US FDA has recently updated its guidance on COVID vaccine licensure, with specific references to platform technology and immunobridging [49]. Studies on the disease for which the vaccine is being developed should be assessed to determine the quality of evidence to support the clinical relevance of the immune marker [50]. The studies should be sufficiently stringent to mitigate against erroneously concluding vaccine effectiveness. 

## 7. Applying the Platform Technology Approach to a Wider Range of mRNA Products 

**Figure 1:**
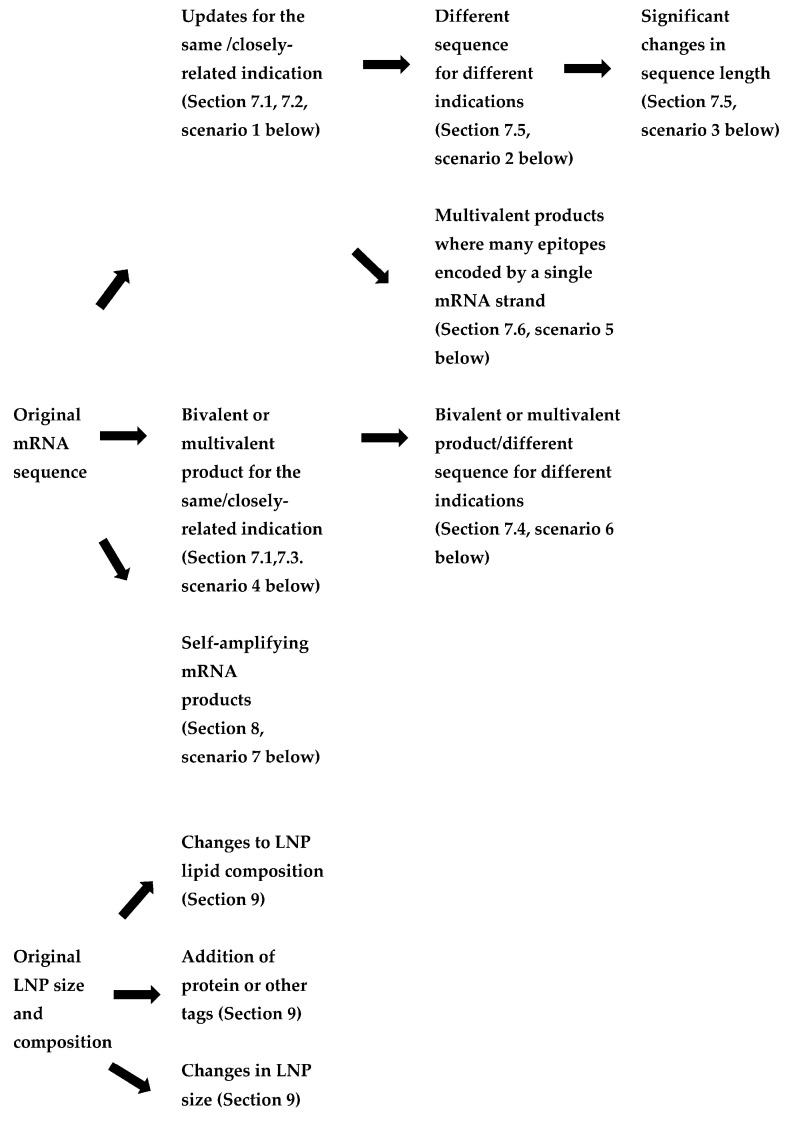
Flowchart of the review—hierarchy of potential changes to mRNA products.

As mRNA technology is applied to a wider range of vaccines and therapeutics, the nature of products is also becoming more diverse. In some cases, the products are becoming more structurally complex. It is possible to construct a potential hierarchy of mRNA products, depending on the level of structural and functional similarity with products that have already been developed and/or passed or undergoing regulatory review. A hierarchy of changes to an original mRNA product could be envisaged (Figure 1, Table 2). For mRNA coding and non-coding sequences, this could include:

1. Updates to mRNA sequence for the same or closely related indication (to enable improvement to a vaccine or therapeutic and/or for a vaccine against a viral variant). In these cases, changes to both the coding (open reading frame) and non-coding sequences could be made.
2. The use of a different mRNA sequence for a vaccine or therapeutic treating different indications within the same family of products (e.g., respiratory viruses, metabolic diseases).
3. Significant changes in mRNA sequence length, but targeting the same disease (for example, a shorter mRNA encoding a subunit or epitope rather than the full protein), which affect its stability and delivery.
4. Monovalent products (single mRNA sequence) vs. bivalent or multivalent products, where the encoding mRNAs are on separate strands. These may either be for variants or seasonal updates to an ancestral vaccine or products targeting diseases which require multiple proteins to be expressed.
5. Products where multiple short antigenic peptides are encoded in a single sequence where each set of selected epitopes are specific to an individual (e.g., individualised neoantigen therapies).
6. Therapeutic products addressing different targets of the same metabolic pathway (e.g., a group of rare diseases caused by different faulty enzymes of the same cellular pathway).
7. Self-amplifying mRNA products, where the replicon mRNA included both mRNAs for enzymes involved in amplification as well as the coding sequence for the target antigen.

### 7.1. Experience with COVID Vaccines

There is already significant global experience with this approach, in the development and regulatory evaluation of variants for COVID vaccines, where many aspects of the platform technology approach were utilised. The variants involved the first example listed above, namely, updates to the mRNA sequence for the vaccine against the same indication. The initial COVID-19 mRNA vaccines used during 2021 contained a single mRNA sequence encoding the SARS -CoV-2 spike protein, and with overall lengths [51] of 4284 nucleotides (Pfizer-BioNTech BNT162b2) and 4004 nucleotides (Moderna mRNA-1273) and similar, but not identical, LNPs. 

For example, in the development of the BA.1 variant Moderna vaccine, the immunogenicity of elasomeran (mRNA-1273, vaccine to the ancestral virus with the D614G mutation), imelasomeran (mRNA-1273.529, vaccine to the BA.1 variant), and elasomeran and imelasomeran combined (mRNA-1273.214) were determined in two animal models (mice and non-human primates). Immunogenicity data in non-clinical studies demonstrated the bivalent vaccine combination (after primary vaccination with elasomeran) resulted in greater cross-variant neutralisation and cross-reactive B-cells in the draining lymph nodes against the ancestral and Omicron BA.1 and BA.2 (sub)variants than boosting with the ancestral vaccine. Protection studies were limited to primary immunisation with the ancestral monovalent vaccine and boosting with either the monovalent vaccine or the bivalent vaccine. Protection by an imelasomeran booster dose against the Omicron BA.1 subvariant was demonstrated in mice and non-human primates. A booster dose of imelasomeran after primary immunisation with elasomeran reduced viral loads in upper and lower respiratory tracts, pro-inflammatory cytokines, and lung pathology. 

Repeat dose toxicity studies were conducted in rats. Human safety, reactogenicity and immunogenicity data were developed comparing boosting with the bivalent versus the ancestral monovalent vaccines with non-inferior neutralising antibody responses as the endpoint. 

With the subsequent ancestral/Omicron BA 4.5 bivalent vaccine from the same sponsor, non-clinical studies of immunogenicity and protection were only required to be performed in a single species (mice), and a similar approach to clinical data generation was used as for the BA.1 variant (although the EU and US regulators did not require human data to be submitted prior to authorisation). Apart from the standard CMC data, expression and identity assays were required, with Expi293 cells transfected with elasomeran and davesomeran and separately identified. No toxicity studies on the BA 4.5 bivalent vaccine were required to be submitted, since the new mRNA used the same backbone and manufacture platform as elasomeran and there are no changes to vaccine formulation except for the additional mRNA. 

The monovalent vaccines to the XBB 1.5 variant were evaluated as regulatory variations to the original vaccines rather than as the subject of new applications for provisional registration. Using the Pfizer-BioNTech XBB 1.5 vaccine as an example, no human clinical data was submitted to the TGA, and the efficacy of the product Comirnaty Omicron XBB.1.5 was inferred from efficacy data of the prior Comirnaty (tozinameran) vaccines. Preclinical immunogenicity studies involved one species (mice) and were based on familiarity with the platform; neither protection studies, genotoxicity, nor carcinogenicity studies were performed. In Australia, the *Therapeutic Goods Act 1989*, which determines the regulatory framework, required that clinical trial data be submitted for the review of the BA4.5 mRNA vaccines for provisional approval; clinical data were not required for regulatory evaluation of the same products in the USA and Europe.

Therefore, as the experience with each platform grew and understanding of the impacts of sequence differences between variants [52], regulators both in the EU and internationally required progressively less data on each cycle of vaccine against newer SARS-CoV-2 variants, such that for the most recent approvals (XBB.1.5), the process was more analogous to that used for regulatory review of updates to seasonal influenza vaccines [53].

### 7.2. Updates to mRNA Sequence for the Same Indication 

One major advantage of the mRNA vaccine platform over alternative vaccine platforms is that they are very readily and rapidly updated to develop vaccines against variants. Vaccines against variants are needed for seasonal influenza vaccines, but may also be needed for some other vaccines against other single-stranded RNA viruses for which mRNA vaccines are under development. These include HIV-1 (human immunodeficiency virus-1), HTLV-1 (human T-cell lymphotropic virus 1), HCV (Hepatitis C virus), RSV, Ebola, West Nile fever, Dengue, Zika, and Chikungunya viruses [54]. As mRNA technology evolves, changes to coding and non-coding sequences can also be made to improve protein expression or stability, reduce reactogenicity, or restrict expression to desired cell types through incorporation of miRNA control elements. Similarly, modifications may be made to the coding regions of mRNAs expressing monoclonal antibody light or heavy chains [55,56] to increase antibody binding affinity or slow metabolism of the expressed proteins.

Vaccine developers should be able to leverage information in their regulatory submission such as existing analytical, nonclinical, and clinical data on comparable changes made to an mRNA sequence from a platform product that has a shared mechanism of action and indication and is using the same LNP and route of administration. 

***Manufacturing and quality data.*** A sponsor could reasonably apply the same manufacturing process controls, analytical test methods, and qualifications of those analytical test methods to evaluate manufacturing process controls for all stages of mRNA-LNP product manufacture. Prior knowledge gained from product characterisation studies may also be relied upon because some updates to the mRNA sequence would be unlikely to fundamentally change the product in terms of its quality or manufacturing characteristics. An example is the addition of a short non-coding unique sequence identifier to enable analytical identification of each specific mRNA.

Some limited changes to an existing platform product’s mRNA sequence may not result in new product quality attributes. The steps to manufacture the modified mRNA sequence would also not change. With any update to the RNA sequence, there is also a corresponding change to the sequence of the DNA plasmid. In order to demonstrate that the quality attributes of the existing mRNA product and the updated product are highly similar, a manufacturing and analytical comparability assessment should be proposed to the regulator and conducted by the manufacturer. 

Additional data requirements could include:

- Information on the sequence accuracy for the new DNA template and mRNA product. However, the same manufacturing process, controls, and analytical methods are applied for the new cell bank generating the new plasmid containing the varied sequence.
- Demonstration that the levels of expression of the new mRNA are comparable.
- Demonstration that the process and product-related impurities are comparable.
- If changes affect the coding regions, identity testing and analysis of expression levels of the altered protein is also required.

***Preclinical and clinical data:*** The comparability assessment of the existing and updated product can help determine the relevance of the non-clinical and clinical data package to the updated product. If only relatively small updates are made to the mRNA sequence, most of the nonclinical profile of an updated mRNA-LNP would not be expected to change. New nonclinical data could be limited to single-species bridging studies (typically mouse) for any relevant toxicology, pharmacodynamic, or pharmacokinetic endpoints.

In cases of well-established pharmacodynamic endpoints, it may be possible to rely upon surrogate endpoints or correlates, such as neutralising antibodies, rather than requiring clinical trial data on actual efficacy against diseases for new vaccines [57]. For seasonal variant updates, no human data may be required by regulatory agencies. The platform approach can also be used to justify the proposed dose for a new vaccine that is utilising the same route of administration. 

### 7.3. Bivalent Products for the Same Indication (e.g., Certain COVID-19 Vaccines) 

***Manufacturing and quality data.*** These are largely treated in the same way as monovalent products, with some additional process development and validation required. It is critical to ensure that each mRNA is manufactured consistently, especially where two separate mRNA-LNP drug substances are manufactured separately and mixed to prepare the drug products. The identity assay should be able to distinguish each variant vaccine type in the final drug products. The potency assay should reliably ensure that each component is measurable separately.

***Pre-clinical and clinical data.*** Assays for immunogenicity must be able to assess either the contribution of each component in the vaccine to the immune response or to each target antigen. Failing this, they should at least demonstrate that the bivalent vaccine has a non-inferior response to the variant antigen than the original monovalent vaccine. With potency assays, assays for immunogenicity should be specific to the variant, noting that correlates of protection may not be as well established for variants as for the ancestral form of the disease. Potency in vivo is also dependent on the uptake of the mRNA-LNP, which should be able to be inferred from the mRNA-LNP for the original virus strain. 

### 7.4. Other Bivalent or Multivalent Products

Such mRNA platform products include the following:

- Two or more existing mRNA sequences against multiple variants or in combination products (e.g., COVID-19 and influenza mRNAs).
- A combination of existing and new mRNA sequences.
- Two or more new mRNA sequences, e.g., for a vaccine, where use of a range of antigens is considered important to mount a broad immune response [28], or for a therapeutic where both subunits of an enzyme must be expressed to regain function.

The regulatory data requirements for a new bivalent or multivalent product will vary depending on whether the mRNA sequences have been part of mRNA platform products that have already had regulatory approval and/or target the same or closely related indications as preceding mRNA platform products.

***Manufacturing and quality data.*** There are two broad approaches to manufacturing: co-formulation within a single LNP, or admixtures of two mRNA-LNP complexes (as was used for bivalent COVID-19 vaccines). Comparative mouse studies for mRNA influenza vaccines have demonstrated that both approaches can potentially be effective [58]. 

For admixtures, there is no change to the upstream manufacturing processes (DNA template, mRNA synthesis, and LNP synthesis), while for co-formulation, assurance will be required around encapsulation efficiency. There are several important CMC considerations, including the need to assess the potential interference or interactions of individual mRNA components on the target molecule expression, purity, potency, and stability. It is also important to characterise the individual mRNA elements to optimise the delivery stoichiometry and assess the impact of multiple mRNA sequences encapsulated by LNP on purification methods and final drug product sterile filtration. Some of these interactions could include formation of RNA-annealed structures that could trigger innate dsRNA sensing pathways or miRNA processing. 

When combining two or more existing mRNA sequences, a sponsor can rely on previously established manufacturing processes and analytical control strategies for all manufacturing stages. For two or more “new” mRNA sequences, the sponsor may be able to apply manufacturing process and analytical control strategies from the well-established mRNA platform that is shared by those products within the same product family. 

For admixed bivalent and multivalent vaccines, information on the mixing step is critical. Combining two or more mRNAs with LNP may impact the process and analytical control strategies and require adjustment; there may be a need to develop/validate multiple mRNA manufacturing and analytical methods. The identity and quantity of each mRNA should be established (confirmation by sequence analysis is required). Identity tests are also required for the expressed product of each mRNA in a multivalent product and ensure that the ratio of each expressed product is as intended. There may be a need to determine through sequencing whether there are effects on miRNA and cellular RNA expression.

***Pre-clinical and clinical data.*** For a product that combines existing products., e.g., COVID-19 and influenza, the individual nonclinical and clinical data packages can be leveraged in their entirety as long as appropriate bridging studies are conducted with the new bivalent product to demonstrate their continued relevance. Nonclinical pharmacology, toxicology, and biodistribution data regarding mRNA-LNP components that are common to the other products from the platform should be able to be leveraged. A single species-bridging study could be required to demonstrate that the biodistribution and toxicological characteristics of the components are maintained. 

The regulatory submission must also include a scientific and clinical rationale for the combination product. Non-clinical and clinical safety data from a monovalent product formulation can support the clinical development of a multivalent formulation for the same or related diseases where the mRNA content and mRNA/LNP ratios are similar, total mRNA dose is similar, and the same LNP is used. Investigation of the potential for interference between products, potentiation of either product’s effect, and/or potential for new safety signals must also be conducted. 

For the combination of vaccine products against different diseases (e.g., influenza and COVID-19), new clinical trial data are usually required by regulators to demonstrate that the efficacy and safety of the combination support the new indication. Prior translation of non-clinical to clinical findings in existing products would support leveraging existing safety or dosing information if new product demonstrates similar non-clinical findings.

For two or more “new” mRNA sequences new clinical and non-clinical data will be required in the regulatory submission. Irrespective of whether sequences are “new” or have been used in other products, both non-clinical and clinical data modules should consider the impact of the total dose of the mRNA in cases of a combination vaccine to avoid reactogenicity and other adverse events. If there is a corresponding monovalent vaccine, the rule of thumb is to evaluate no more than the maximum dose for that vaccine, unless pre-clinical and clinical experience determines that that dose is inadequate from an efficacy standpoint.

### 7.5. Different mRNA Sequences for Different Indications Using the Same LNP and Route of Administration

Examples of such products include:

- Vaccines against infectious diseases when there is wide genetic or antigenic diversity with an infection—e.g., HIV mRNA candidate vaccines with a single sequence co-expressing two antigens [20], mRNA seasonal influenza vaccines [37], or where multiple antigens may be required, e.g., for norovirus vaccines. For some antigens, such as cytomegalovirus, the candidate mRNA vaccine is reflective of the complex viral structure [20].
- Where expression of multiple enzyme subunits is required to regain function in a treated patient, e.g., propionic acidemia [59].
- mRNAs that independently deliver heavy and light chains of a therapeutic or prophylactic monoclonal antibody [28,55,56,60].
- Oncology mRNA vaccines encoding several tumour-associated antigens or that express a mix of cytokines that mediate tumour regression [61].
- When the vaccine needs to cover more than one species of a pathogen (e.g., *Borrelia* in Lyme disease).

In these cases, applicants should usually be able to leverage information in a regulatory submission in the application of the same mRNA platform where the product is being updated with a new mRNA but is formulated within the same LNP within the same larger product family. The applicant will need to consider whether there is a possibility that the new sequence change could impact the process and analytical control strategies and require new specifications or additional characterisation. 

***Manufacturing and quality data.*** The new mRNA sequence may only require minimal changes to the manufacturing process. The applicant should be able to apply the same manufacturing process controls, test methods, qualification of test methods, specifications, and proven acceptable ranges for the LNP manufacturing stage of the process [62]. The manufacturing process controls, analytical test methods, and qualification of those analytical test methods for the other manufacturing stages (e.g., DNA template manufacture, mRNA encapsulation) will be consistent with the preceding mRNA platform product. This is especially true for those from the same family of products (e.g., prophylactic vaccines for a group similar or related viral pathogens or endogenous intracellular liver enzymes). However, the sponsor should provide evidence that the defined manufacturing process controls for the reference product are still applicable. Identity testing is molecule-specific, and the expression test will require method qualification for a new mRNA sequence. 

***Pre-clinical and clinical data.*** If a new indication is proposed, even if the new product consists of a combination of existing mRNA-LNP products, regulators will require additional nonclinical and clinical data to demonstrate safety and effectiveness. Nonclinical pharmacology, toxicology, and biodistribution data regarding mRNA-LNP components that are common to other products from the platform should be able to be leveraged. However, a biodistribution study with the new mRNA sequence, along with reference to existing biodistribution data using the same LNP with other similar intended uses, may be required. 

The type of encoded protein (endogenous or exogenous, intracellular or extracellular) and the cellular or tissue target for mRNA translation will have significant influences on potential toxicology [42]. The ability to bridge from earlier data will be more limited if the type of encoded protein changes. The amount of non-clinical safety and toxicology information will be guided by the level of knowledge on the pathology of the disease being targeted. For a new disease, non-clinical bridging studies will be required to demonstrate the nonclinical biological effects of the new product are similar to the existing products. 

Prior translation of nonclinical to clinical findings in existing products may support leveraging of dosing information if the new product demonstrates similar nonclinical findings. Justification of appropriate dosing is required, and bridging alone may not be sufficient to support the anticipated effective clinical dose. 

For vaccines or therapeutics against a new disease, a full clinical data package will be required. Typical clinical programs for novel products require initial demonstrations of safety, pharmacologic effects, as well as an exploration of dose findings through Phase 1/2 studies, followed by a demonstration of safety and efficacy with well-controlled Phase 3 trials [63]. When a new related product relies upon similar mechanism(s) of action or otherwise acts on similar pathophysiological pathways as existing approved products, previous demonstrations of clinical efficacy or an effect on a biomarker may be applicable to the new product. New indications for which a biomarker or agreed surrogate outcome measure does not exist will require data on direct assessment of clinical efficacy outcomes.

In some cases, significant changes to the mRNA length or structure could potentially require larger LNPs, or LNPs of different composition [64] and evidence would need to be submitted to determine how bridging could be used. Information is needed on product characterisation, in-process controls, including the test methods and their qualification. The regulatory submission must include a scientific and clinical rationale for the new product. Nonclinical pharmacology, toxicology, and biodistribution data regarding mRNA-LNP components that are common to the other products from the platform should be able to be leveraged, although new data relating to the impact of the new sequence and new LNP will be required. A bridging study would be required to demonstrate that biodistribution and toxicological characteristics of the components are maintained. 

### 7.6. Multivalent Products Where Many Epitopes Are Encoded by a Single Strand of mRNA

In other cases, the different sequences will be included in a single mRNA strand (e.g., cancer vaccines encoding multiple tumour neoantigens) [65,66]. While the overall length of the mRNA construct may in fact not be greater than mRNAs expressing other single antigens, with multiple mRNA sequences being aligned end-to-end and translated into a longer polypeptide, it is important for the sponsor to design the mRNA to minimise the potential formation of new, unintended antigenic sequences (epitopes) that could potentially arise at the junctions of two peptides. Preclinical and clinical evidence may be required so that immune response to these additional epitopes do not develop.

For concatenated products, any changes to synthesis protocols (such as the use of mRNA block methods to synthesize concatenated mRNAs) should be identified and documented in the submission. The extent to which data from other mRNA platforms may be used will depend on the extent of changes to the sequence and/or LNP delivery. If the concatemer product are composed of mRNAs encoding a number of short peptide epitopes (e.g., as in cancer neoantigen immunotherapeutics), some platform data may be leveraged if the length of the mRNA would be comparable and the LNP and composition payload are similar to existing licensed products. 

## 8. Considerations for Self-Amplifying mRNA Products

Conventional mRNA (non-amplifying) approaches have overwhelmingly been the main technology used to date. Commercial experience with sa-mRNA products is rather limited, with only two SARS-CoV-2 sa-mRNA-LNP vaccines with regulatory emergency authorisation or approval in India and Japan, respectively [67,68]. Several other sa-mRNA vaccines for SARS-CoV-2 and targets are in active development against a range of viral, parasite, and bacterial antigens and oncology targets [69,70,71,72], including more than a dozen in clinical trials [73]. The sa-mRNA approach may also enable mRNA technologies to deliver monoclonal antibodies for passive immunisation, as comparatively low doses of mRNA can enable expression of the relatively large concentrations of protein that may be required [62].

The sa-mRNA vaccines differ from conventional mRNA vaccines in that, as well as having an open reading frame coding for the antigen of interest, have an upstream initial open reading frame that expresses viral replicase proteins that activate high level replication of the therapeutic mRNA product after transfection in vivo. These features naturally make sa-mRNA more complex than non-amplifying RNA. The doses of sa-mRNA being clinically trialled are typically lower than for mRNA. While conventional vaccines contain a number of nucleotide modifications, such as replacement of uridine with N-1-methyl pseudouridine. In contrast, sa-mRNA vaccines must use uridine and cannot optimise RNA sequences interacting with the viral replicase protein. The trade-off in reactogenicity due to use of non-modified nucleosides on one hand versus the use of lower total mRNA doses on the other is an issue for optimising doses in sa-mRNA studies. 

mRNA platform technology considerations can certainly be applied to sa-mRNA products, although, because of the differences listed above, in some areas, they would be required to be treated as a ‘sub-platform’ within the platform. The general dossier requirements described above will apply. Regulatory submission for sa-mRNA products will also require specific information on the following issues.

***Manufacturing and quality data.*** Information on the origin of the self-amplifying replicon genes must be provided, together with details of the organisation and sequence of, and modifications to, both the replicon RNA including the gene of interest. Alphavirus-based replicons contain a separate open reading frame that encodes all the replicase polyprotein upstream of an internal sub-genomic promoter supporting the expression of the antigen sequence, while flavivirus-based replicons, the replicase proteins are encoded in a single open reading frame downstream of the antigen encoding sequence typically expressed as a unimolecular polyprotein [74]. 

For sa-mRNA, where the mRNA encoding the replicon and the mRNA encoding the target antigen are encoded on different mRNA strands, it is usual for the two mRNAs to be encapsulated within the LNP together so that they will consistently be taken up by the same cell following administration [70,71]. If the two RNAs are encapsulated separately, this must be documented, and the regulator may require data on co-expression. As sa-mRNA can be up to 12,000 nucleotides in length, manufacturing is more challenging. At each stage of the manufacturing process, information on purification and tests for the presence of fragments are required, given the susceptibility to degradation of long mRNA sequences. This is a particular issue with alphavirus replicases, as they are encoded by a large 8–9 kb sequence (encoding four non-structural proteins—initially made as a polyprotein and a sub-genomic promoter). Unless comparing the product to another sa-mRNA product, detailed stability studies may be required due to the size and complexity of sa-mRNA.

***Preclinical and clinical data.*** The total dose of the sa-mRNA product requires assessment and documentation, along with impact on innate immune expression. Because sa-mRNAs are not able to utilise pseudouridine nucleoside modification, the unmodified sequence is likely to provide greater type 1 interferon responses [75], although they are potentially offset by the anticipated lower mRNA doses of these products. 

Pharmacokinetic studies on the persistence of replicons (and expressed antigen) and the duration of immune response may be required. Unless the manufacturer is bridging from another sa-mRNA product, a larger number of toxicological and biodistribution studies will be required. Genotoxicity and pregnancy data may also be required, unless the comparison is with another related sa-mRNA platform.

Unless the manufacturer is bridging from another sa-mRNA product, a full clinical module to the dossier submission would be required, with detailed information on the nature of the immune response to the product in humans, clinical efficacy, reactogenicity, and safety. Human pharmacokinetic and toxicology studies will also be required, particularly with information on the persistence of both the sa-mRNA and its components and the proteins expressed by the target mRNA.

## 9. Lipid Nanoparticle Variations

This review focusses on (unmodified) LNP-based delivery systems for mRNA, as these predominate both in the vaccines that have been commercialised or are in phase 2 and 3 clinical trials (and are thus most likely to be commercialised soon). They are composed of a combination of different lipids, such as ionisable lipids, phospholipids, cholesterol, and/or polyethylene glycol (PEG)-lipids [76,77,78]. There has been increasing interest in modified LNPs, allowing for more robust translational output; customisation of the lipid’s outer layer potentially allows for greater targeting of desired cell types and can affect the pharmacokinetics of the LNP-mRNA. 

Data on the formulation of the LNP and encapsulation of the mRNA by the LNP are required in the original regulatory submission for the first platform product from each sponsor [79,80]. Lipid nanoparticles used for mRNA delivery can vary in size, size distribution (polydispersity), and lipid composition of lipid nanoparticles (from the same sponsor). In addition, surface modification of LNPs, including the addition of protein tags [81], have been trialled to alter pharmacokinetics or biodistribution, and there are several other mRNA delivery systems in preclinical and clinical development. 

There has been continuous evolution in the choice and composition of lipids used in LNPs over some years [82,83] to increase the in vivo delivery and thus the efficacy of LNPs and to reduce toxicity. The differences in LNP composition between the approved mRNA vaccines is one reason why the products must be considered to be different mRNA-LNP platforms. However, if the LNPs used in several of the products in clinical trials for an individual sponsor are the same as or reasonably similar to that in the authorised the COVID-19 vaccine and will facilitate regulatory consideration of them as being part of the same platform.

More recently, one of the main drivers for modification of lipids in LNP is to achieve selective organ targeting [83,84]. Modification of phospholipid/cholesterol ratios also influences circulation times of LNP. Other strategies, such as mannose labelling or conjugation with specific antibodies have been employed to increase the dendritic cell targeting and lung expression, respectively, of mRNA-LNP products. Approaches for varying the composition of the LNPs to target the biodistribution of the mRNA in a range of cancers, including to leukocytes, the liver, spleen, and lung, have been reviewed [77]. 

***Manufacturing and quality data.*** If the LNPs of the same size and composition are used for subsequent products, even if the mRNA codes for a quite different antigen and the product has very different indications, the LNP manufacturing encapsulation data can be extrapolated for regulatory purposes. If manufacturing methods or the size or composition of LNP changes, then suitable bridging data will be required in the submission for that product. If LNPs are augmented with other components such as particular proteins (including or excluding antibodies), detailed manufacturing data will in particular be required for these types of LNPs.

***Preclinical and clinical data.*** Slight compositional changes (i.e., changing ratios of existing lipids, changes in buffers not impacting the characteristics of the LNPs) should not need new toxicological data. However, full toxicological data will need to be submitted for regulatory evaluation if a novel lipid is used. If LNP formulations are modified to increase delivery of mRNA to specific cell types, regulators will request data on the information on influences on protein expression and its localisation. Clinical data requirements relating to changes in LNP composition, size, or functionality will be informed by preclinical studies, as well as the nature of the disease or condition being treated.

## 10. Particular Considerations for mRNA Therapeutics

Several mRNA-LNP therapeutics are under clinical development [23,85,86,87,88,89,90], including oncology immune-therapeutics for a range of cancers including melanoma and other skin cancers, lung, cervical, breast, and ovarian cancers, liver and gastric cancers, pancreatic cancer, bladder cancer, prostate cancer, and head and neck cancers. mRNA protein replacement therapeutics are being developed for a range of genetic diseases including cystic fibrosis and rare metabolic diseases, type 2 diabetes, cardiovascular disease, and other fibroses [91,92]. There are also potential safety advantages for mRNA therapeutics over gene therapies because there is no genome integration or permanent modification of the genome. mRNA therapeutics bring manufacturing advantages compared with the production of recombinant proteins in that much smaller quantities are typically required. 

Therapeutics are anticipated to require chronic, repeat administration at (say) monthly intervals, compared with (at most) annual booster shots of certain vaccines. While EMA and FDA usually do not require pharmacokinetic studies for vaccines (although distribution studies may be needed for new vaccine formulation, adjuvants or routes of administration [42]), it is anticipated, however, that more detailed pharmacokinetic studies will be required for therapeutic mRNAs. Particularly for non-amplifying mRNA products, it is also expected that many therapeutic applications will require higher doses of the mRNA than vaccine applications [88]. Some of the clinical trials of mRNA cancer products have involved nine or more doses. These factors are likely to lead to greater accumulation of mRNA, mRNA fragments and translated proteins, and peptides following administration than with vaccines, as well as greater levels of LNP degradation products.

Therapeutic mRNA products may also require different LNPs, depending on whether systemic (usually intravenous), intra-tumour, or inhaled dose forms are required, and with attempts to improve organ targeting. In contrast to the fate of intramuscularly administered mRNA vaccines, a significant proportion of the mRNA and lipid from intravenously administered mRNA therapeutics passes through the liver. Some therapeutics are also likely to have multiple mRNAs in each product. While the therapeutic purpose—treatment rather than prevention of a disease—is different for a mRNA-LNP therapeutic than a vaccine, the technology used for their manufacture is identical or at least highly similar. Therefore, platform technology considerations of manufacturing and product characterisation can be directly applied and included in the regulatory submission. In certain cases, therapeutics development may bring with it specific mRNA optimisation and delivery challenges. Alternative packaging systems to LNPs are being utilised to improve targeting [88]. 

***Manufacturing and quality data.*** If the mRNA characteristics and LNP composition and size are similar to that used in a vaccine product from the same manufacturer, there are a number of platform aspects that could reduce the need for generation of new data. Data on the duration of protein expression in vitro will also be required. 

***Preclinical and clinical data.*** The differing nature of each therapeutic, e.g., cellular targeting, dosage, number of doses, means that sponsors should be strongly encouraged to discuss non-clinical regulatory requirements during development of these products with regulatory agencies prior to submission. More extensive requirements for pharmacokinetic, repeat-dose toxicology, and genotoxicity data are likely to be required for a mRNA therapeutic [87,88], including on the possible accumulation of lipids from LNPs in mRNA therapeutics. 

Delivery to target organs will need to be documented in the regulatory submission. More frequent dosing could lead to the accumulation of lipids from nanoparticle carriers, and unwanted immune activation may limit efficacy as mRNA potentially recognised by the body as viral RNA, so biodistribution studies will be required for mRNA therapeutics [42]. The duration of protein expression [93] and its relationship with efficacy of the product in animal models of the target disease should be documented in regulatory submissions. Data on immune activation if higher and/or repeat doses are required, as it may limit efficacy of the therapeutic and lead to adverse events. 

It is anticipated that regulators will require a comprehensive package of clinical data, particularly for the first mRNA therapeutic in each drug class. This should include justification of the dose used, and how it has been extrapolated from preclinical studies [61]. Detailed human pharmacokinetic data, for both the mRNA/s, fragments, protein products, and LNPs and their degradation products is required. Routes of administration may differ from the typical intramuscular or intradermal route used for vaccines and include intravenous, subcutaneous, or inhalation. Efficacy data from clinical trials should include evidence on the duration of protein expression and its relationship with the efficacy of the product. Detailed information on adverse events and reactogenicity, particularly in relation to repeat doses, will be required.

## 11. Implications for Personalised Medicine and Rare Disease mRNA Products 

One of the most rapidly growing areas of mRNA product development is for cancer vaccines [61,87,94]. Immunisation against cancer-related antigens aims to induce an effective immune response against the tumour and utilises mRNA sequences in the treatment that have been synthesised to encode either tumour-associated or tumour-specific antigens. In a number of cases, the mRNA product has been administered with a checkpoint inhibitor as the mRNA product is designed to mount a cancer specific T-cell immune response to complement the checkpoint inhibition therapy in what are often highly immunosuppressive tumour micro-environments. 

Tumour-specific neoantigens are a form of personalised cancer immunotherapy, and each collection of neoantigen sequences is tailored to the individual profile of the candidate [65,66,95]. A set of corresponding mRNAs can then be quickly synthesised for use in an mRNA product to mount an immune response against the tumour. This innovative approach to mRNA-LNP product development breaks new ground from a regulatory standpoint. Regulators will be required to adapt their manufacturing oversight to review facilities that conduct simultaneous very small-scale manufacture of a product under GMP conditions.

Because each batch of an individualised product has unique properties due to changes in the selection of neoantigen epitopes for incorporation in the mRNA sequence open reading frame, the standard approach to demonstrating comparability of each product that encodes a new sequence is not possible. This is because every patient’s product comprises a unique set of sequences. Regulatory approaches will require adaptation to accommodate this. 

Three broad approaches could potentially be used:

- The manufacturing stream for a given individualised neoantigen product could be split from a single DNA template starting material throughout two parallel manufacturing processes to produce a pair of batches, which can then be compared at the range of process validation steps.
- Design a set of sequences that encompasses the extremes of potential patient-specific sequences and assess the reliability of expected versus actual product characteristics.
- Evaluate manufacturing updates at the process level, including updates to genome sequencing and bioinformatics.

Another example of tailored therapeutic use of mRNA technology is for rare diseases [91,92,96]. Many of these diseases are due to mutations in enzymes from various metabolic pathways, which can be compensated for by providing the native, fully functional enzyme through an mRNA. Many of these diseases are extremely rare and almost unique to some individuals. Rather than considering every single mRNA therapy encoding for a single enzyme as a separate product, from a regulatory standpoint these could be grouped into a single “umbrella” product that would cover a range of enzymes pertaining to the same metabolic cycle. Similarly to the individualised cancer therapies, approaches to streamline the regulatory process and leverage the platform knowledge base would enable a much faster access of these therapies to the patients in need.

## 12. Conclusions

Experience with mRNA COVID variant vaccines has shown that use of a platform approach can streamline regulatory review without exposing the public to safety risks or efficacy concerns, increasing confidence in mRNA technology. With a number of other products either already submitted for regulatory review or in late-stage clinical trials, and the mRNA and LNP design of many of these products being heavily based on the COVID vaccines from the same manufacturer, it is feasible to utilise the platform approach for these products. The comparability and bridging studies required will depend on the extent of difference to the original vaccines. 

The concept of facilitating medicine development and regulatory review based on “prior knowledge” has appeared in regulatory guidance and influenced regulatory thinking for many years. Vaccine development has also centred around platforms based on the technology used in their manufacturing for some time [97]. mRNA product technology is highly amenable to the establishment of platform processes. mRNA technology readily can be considered an “end to end platform for vaccine production” [16], given the common approaches used for target design, mRNA synthesis, mRNA-LNP drug substance, and drug product preparation. Other vaccine and biological development and manufacturing requires much more process customisation, particularly of downstream purification of the recombinant protein. It is also important to treat the set of analytical techniques used throughout the manufacturing process and product lifecycle as a platform [15] so that the analytical processes can test quality attributes of a range of different products without major changes to test procedures. 

Establishing a consensus definition among regulators and developers of platform technology specific to mRNA vaccines is a critical first step. There is reasonably good alignment between the platform concepts described by major global regulators and the WHO, and utilisation of the platform approach, including pragmatic use of comparability and bridging studies, was a feature of the evolving experience during the development and regulatory review of COVID-19 mRNA vaccines. 

Platform technologies can potentially be applied to the development and review of a wider range of mRNA products, including what new data and analytical techniques may be required. Agreed regulatory approaches are needed urgently to avoid unnecessary development costs and timeframes and delays to consumer access to other important mRNA vaccines and therapeutics, several of which are in advanced clinical trials or currently under regulatory review.

## Figures and Tables

**Table 1:** mRNA products commercialised or in clinical trials.

Sponsor	Commercial or Submitted for Regulatory Review	Phase 3	Phase 2	Phase 1/2	Phase 1
Moderna (https://trials.modernatx.com/search-results/) (accessed 1 May 2024)	Various SARS-CoV-2 vaccines mRNA1345—Respiratory Syncytial Virus	mRNA1647—Cytomegalovirus (women) mRNA1010—Seasonal influenza mRNA1083—Seasonal influenza—COVID combination mRNA4157—Melanoma, personalised individual neoantigen therapy (with pembrolizumab)	mRNA1647—Cytomegalovirus (extension trials) mRNA1893—Zika virus	mRNA1769—Smallpox/ monkeypox mRNA1608—Genital herpes mRNA1975/1982—Lyme disease mRNA3210—Phenylketonuria mRNA1468—Shingles	mRNA3745—Glycogen storage disease mRNA2752—Lymphoma, Triple negative breast cancer mRNA0184—Chronic heart failure mRNA 1195—Epstein–Barr virus mRNA1365—RSV plus human metapneumovirus mRNA 4157—Personalised neoantigen therapy mRNA1403—Norovirus mRNA1653—Metapneumovirus and parainfluenza mRNA1944—Chikungunya mRNA6231—Autoimmune diseases
BioNTech (in some case with partners, e.g., Pfizer, Genetech and Genmab) (www.biontech.com/int/en/home/pipeline-and-products/pipeline.html) (accessed 1 May 2024)	A range of COVID-19 vaccines (with Pfizer)	BNT161—Seasonal influenza BNT162b2+ BNT161 COVID-19- Influenza combination	BNT111—Advanced, R/R melanoma BNT113—Metastatic/R/R HPV16+ head and neck cancer, metastatic NSCLC BNT116—Metastatic non-small cell lung cancer BNT122 (autogene cevumeran)—Advanced melanoma, advanced colorectal cancer, Adjuvant pancreatic ductal adenocarcinoma BNT162b5/6/7—COVID-19 (ancestral + BA 2)	BNT142—Solid tumours BNT151—Solid tumours BNT165—Malaria BNT166—Mpox BNT167—Shingles	BNT112—Metastatic/localised prostate cancer BNT116—Advanced/ metastatic NSCLC BNT122 (autogene cevumeran)—Solid tumours BNT152+153—Solid tumours BNT163—HSV BNT164—Tuberculosis BNT162b2 BNT162b4—COVID-19
Sanofi (www.sanofi.com/en/our-science/our-pipeline) (accessed 1 May 2024)			SP 0256—RSV and RSV/hMPV bivalent older adults	SP 0273—QIV Influenza SAR441000—Cytokine mRNA for Solid tumours	
GSK (www.gsk.com/en-gb/innovation/pipeline/) (accessed 1 May 2024)			GSK4382276—Seasonal influenza GSK4396687—SARS-CoV 2		
CureVac (www.curevac.com/en/pipeline/) (accessed 1 May 2024)			COVID-19 (with GSK)	Non-small cell lung cancer	Avian and seasonal Influenza (with GSK) Rabies Glioblastoma Solid tumours
Arcturus Therapeutics (https://arcturusrx.com/mrna-medicines-pipeline/) (accessed 1 May 2024)	ARCT-154 COVID-19 sa RNA vaccine (with CSL)	ARCT-2301 Bivalent COVID-19 Ancestral/Omicron BA.4/5 (with CSL) ARCT-2303) Monovalent: COVID XBB.1.5 (with CSL)	Ornithine Transcarbamylase Deficiency		Cystic Fibrosis ARCT-2138—Seasonal influenza Quadrivalent—(with CSL)
Daichi Sankyo (www.daiichisankyo.com/rd/pipeline/) (accessed 1 May 2024)		DS-5670 Monovalent COVID booster (ancestral strain)			
Suzhou Abogen (https://abogenbio.com/en/about) (accessed 1 May 2024)		ABO1020 Monovalent: COVID BA4/5			

**Table 2:** Impacts on quality, pre-clinical, and clinical data requirements for changes to mRNA-LNP products.

Nature of the mRNA Change	Example of Product	mRNA Characteristics	Impact on Quality Data Requirements	Impact On Preclinical Data Requirements	Impact on Clinical Data Requirements
Updates to original sequence for the same or closely-related indication	XBB 1.5 COVID vaccine	Single sequence, similar length as reference mRNA sequence; same LNP Changes to either or both the coding and non-coding regions could be made	**Re-use:** most CMC approaches **Bridge:** Sequence specific analysis, expression and potency assays	**Re-use:** Toxicology, biodistribution Bridge: Comparison of immune responses to reference product New: Possible single species toxicology studies.	**Re-use:** If same indication or for variant (some regulators may want bridging data) New: If indication is different
Bivalent or multivalent product for the same or closely-related indication	Bivalent COVID vaccine, influenza vaccines	Several sequences, homologous to ancestral sequence; same LNP	**Re-use:** CMC for original product **Bridge:** Manufacturing and quality for new sequence Sequence specific analysis, expression/identity, and potency assays that can distinguish products of each sequence	**Re-use:** Preclinical data for original product **Bridge:** Adapt pharmacokinetics, biodistribution, and toxicology analysis for original product **New:** Assessment of contribution of each sequence to the immune response	**Re-use:** Clinical data for original products **Bridge:** Some regulators may require data on a correlate of immunity or protection
Different mRNA sequence for a vaccine or therapeutic treating different indications	Respiratory Syncytial Virus (RSV)	Single sequence, similar length to reference mRNA sequence; same LNP	**Bridge:** CMC for original product if LNP is same; mRNA manufacturing process similar **New:** Sequence specific analysis, expression/identity and potency assays	**Bridge:** Aspects of pharmacology, toxicology and biodistribution data common to reference product **New:** More data will be required for some diseases or if tissue target is different from reference product	**Bridge:** potentially use biomarker if product acts on similar pathways to existing mRNA product **New:** New clinical trial data required to support the new indication
Bivalent or multivalent product, different sequence for different indications	Cytomegalovirus Lyme disease	Multiple sequences, similar length to reference mRNA sequence; Same LNP	**Re-use:** If mRNAs have been in previous products **New:** Encapsulation efficiency (if co-formulated), identities and quantities of expressed mRNA and proteins	**Bridging:** Single species study for biodistribution and toxicology **New:** New non-clinical efficacy data	**New:** New clinical trial data required even for existing mRNAs
Significant changes in mRNA sequence length (including potentially to the non-coding region)	Norovirus Tumour-associated antigens Monoclonal antibodies	May potentially change size and nature of LNP used for delivery	**Bridge:** Manufacturing, analytical and stability studies	**Bridge:** Single species study for biodistribution and toxicology **New:** New non-clinical efficacy (and possibly safety) data	**New:** New clinical trial data required
Multivalent products where many epitopes encoded by single strand of mRNA	Individualised neoantigen therapies	Sequences encoding epitopes included in a single mRNA strand and co-translated	**New:** Require quality and manufacturing approaches that recognise bounds of product manufacture	**New:** Full toxicology, biodistribution and pharmacology data required for representative products, but not all products	**New:** New clinical trial data required for representative products, but not all products
Self-amplifying mRNA products	COVID-19 vaccine Infectious diseases Oncology	Encoding replicon RNA as well as encoding protein of interest A platform within a platform	**New:** Origin of replicon genes, whether encoded on same or different mRNA strand to protein of interest, manufacturing and quality assessments	**Bridge:** Genotoxicity and pregnancy data (if available for another sa-mRNA product) **New:** Full toxicology, biodistribution and pharmacology data	**New:** New clinical trial data required
LNP variations	Oncology Therapeutics	Changes in LNP size, lipid composition or addition of protein or other tags	**Bridge:** If LNP composition similar and only size is changed, or slight changes to composition New: If new lipids or proteins are included	**Bridge:** If LNP composition similar and only size is changed, or slight changes to composition **New:** Full toxicology, biodistribution and pharmacology data otherwise required	**New:** New clinical trial data usually required

## Data Availability

No new data were created or analysed in this study. Data sharing is not applicable to this article.
